# Increased percentages of regulatory T cells are associated with inflammatory and neuroendocrine responses to acute psychological stress and poorer health status in older men and women

**DOI:** 10.1007/s00213-015-3876-3

**Published:** 2015-02-14

**Authors:** Amy Ronaldson, Ahmad M. Gazali, Argita Zalli, Frank Kaiser, Stephen J. Thompson, Brian Henderson, Andrew Steptoe, Livia Carvalho

**Affiliations:** 1 Department of Epidemiology and Public Health, University College London, 1-19, Torrington Place, London, WC1E 6BT UK; 2 Department of Academic Rheumatology, Division of Immunology, Infection and Inflammatory Disease, School of Medicine, Kings College London, Guy’s Campus, London, SE1 1UL UK; 3 Department of Microbial Diseases, UCL-Eastman Dental Institute, London, WC1X 8LD UK

**Keywords:** Regulatory T cell, Psychological stress, HPA axis, Inflammatory response system, Depression

## Abstract

**Rationale:**

The percentage of regulatory T cells (T_Regs_)—a subtype of T lymphocyte that suppresses the immune response—appears to be reduced in a number of stress-related diseases. The role of the T_Reg_ in stress-disease pathways has not yet been investigated.

**Objectives:**

The aim of the study was to investigate the association between biological responsivity to acute psychosocial stress and the percentage of T_Regs_ in healthy older adults. The secondary purpose was to measure the associations between T_Reg_ percentage and psychological and physical well-being in the participants.

**Methods:**

Salivary cortisol and plasma interleukin (IL)-6 samples were obtained from 121 healthy older men and women from the Whitehall II cohort following acute psychophysiological stress testing. Three years later at a follow-up visit, we measured T_Reg_ percentages and psychological and physical well-being were recorded using the Short Form 36 Health Survey and the Center for Epidemiologic Studies Depression Scale.

**Results:**

Blunted cortisol responses (*p* = 0.004) and elevated IL-6 responses (*p* = 0.027) to acute psychophysiological stress were associated with greater T_Reg_ percentage independently of age, sex, BMI, smoking status, employment grade, time of testing, and baseline measures of cortisol and IL-6, respectively. Percentage of T_Regs_ was associated cross-sectionally with lower physical (*p* = 0.043) and mental health status (*p* = 0.008), and higher levels of depressive symptoms (*p* = 0.002), independently of covariates.

**Conclusions:**

Increased levels of T_Regs_ may act as a defence against increased inflammation and may be a pre-indication for chronically stressed individuals on the cusp of clinical illness.

## Introduction

The effects of acute psychological stress on the innate immune system have been widely investigated (Steptoe et al. 2007), and these effects likely mediate, in part, the pathway between stress and disease (Brydon and Steptoe 2005; Pace et al. 2006). However, the effects of acute stress on regulatory T cells (T_Regs_) that modulate the acquired immune system have not been investigated in any detail. T cell dysregulation is present in many auto-immune diseases that are related to psychological stress (Sandberg et al. 2000; Mohr et al. 2004; Straub et al. 2005). However, immunological mechanisms linking psychological stress to exacerbations of autoimmune components of stress-related disorders have not yet been well characterized.

Acute stress leads to transient increases in pro-inflammatory biomarkers such as C-reactive protein (CRP) and interleukin-6 (IL-6) (Steptoe et al. 2007) as part of the normal immune response. This transient and adaptive response may become impaired when stress is chronic. Long-term stress exposure may prime the immune system to produce an exaggerated inflammatory response in times of acute stress (Brydon et al. 2004). Early life stress and high levels of work stress have been associated with heightened IL-6 responses to acute psychological stress (Hamer et al. 2006; Carpenter et al. 2010). Heightened IL-6 responses to acute stress have been found in stress-related diseases (Pace et al. 2006) and have been associated prospectively with increased systolic blood pressure in healthy older adults (Brydon and Steptoe 2005).

Cortisol is the major circulating human glucocorticoid (GC) which serves to inhibit the expression of inflammatory cytokines, such as IL-6, during times of acute stress (Kaltsas et al. 2012). Alterations in cortisol responses to acute stress also are likely to partially mediate the pathway between stress and disease. Prolonged stress exposure over time has been found to affect cortisol responses to acute psychosocial stress, and some studies have found blunted cortisol responses to acute stress challenge in both young and older healthy people who had experienced chronic stress in early life (Miller et al. 2007; Elzinga et al. 2008; Goldman-Mellor et al. 2012). Blunted cortisol responses to acute psychosocial stress have been associated with poor self-reported health (Phillips et al. 2013) and have been observed in a number of stress-related diseases such as major depression (Burke et al. 2005; Miller et al. 2005), fibromyalgia (Wingenfeld et al. 2007), tinnitus (Hébert and Lupien 2007), and in breast cancer survivors with persistent fatigue (Bower et al. 2007). Blunted cortisol responses to acute stress are inversely associated with the production of IL-6 and positively associated with impaired mental health and lower heart rate variability in healthy middle-aged people (Kunz-Ebrecht et al. 2003). Over the past decade, it has become established that one of the major circulating T lymphocytes controlling adaptive immunity, which may be integrated with the stress response, is the T_Reg_ (Kim et al. 2012). T_Regs_ suppress the immune system, thereby preventing pathological immune responses (Sawant and Vignali 2014). These regulatory cells also serve to reduce inflammation through direct effects on T cells and express a number of cytokines that are immunosuppressive in nature, such as IL-35 (Sakaguchi et al. 2009). There are a number of T_Reg_ subtypes with the best understood being those that express CD4, CD25, and FoxP3—the transcription factor required for CD4 + CD25+ T_Reg_ cell development and function (Thompson and Powrie 2004).

T_Reg_ percentages are reduced in a number of stress-related long-term disorders. Decreased T_Reg_ percentages have been found in post-traumatic stress disorder (Sommershof et al. 2009), acute coronary syndrome (Cheng et al. 2008), severe major depression (Li et al. 2010), but not in chronic fatigue syndrome (Brenu et al. 2011). Murine models of atherosclerosis are characterised by decreased T_Reg_ percentages (Ait-Oufella et al. 2006; Mor et al. 2007). Furthermore, both humans and animals with a FoxP3 deficiency and subsequent lack of T_Regs_ can experience devastating autoimmune disease (Rudensky 2011). Loss-of-function mutations in the human FoxP3 gene lead to immunodysregulation, polyendocrinopathy, enteropathy X-linked (IPEX) syndrome—a severe multi-organ autoimmune and inflammatory disorder (Bennett et al. 2001) characterised by decreased peripheral FoxP3+ T_Reg_ levels (Torgerson and Ochs 2007; Barzaghi et al. 2012). This same gene mutation in mice leads to scurfy—an autoimmune disorder characterised by scaly skin, enlargement of the spleen and lymph nodes, and premature death (Ramsdell and Ziegler 2014).

Taken together, these data suggest that a decreased T_Reg_ percentage is characteristic of a number of stress-related autoimmune diseases. However, little is known about how variation in biological responses to acute stress is associated with numbers of T_Regs_. The aim of this study was to investigate the association between biological responses to acute psychosocial stress and the percentage of circulating T_Regs_. We examined the variation in levels of cortisol and IL-6 produced in response to acute psychosocial stress and subsequent percentages of T_Regs_ in healthy older adults. The secondary purpose was to measure the associations between T_Reg_ percentage and psychological and physical well-being in these healthy older adults, as T_Regs_ appear to play a role in many disease states (Miller 2010). Seeing as many stress-related autoimmune disorders are characterised by a decrease in T_Reg_ percentages, we hypothesised that dysregulated biological responses to acute psychosocial stress, i.e. blunted cortisol responses and exaggerated IL-6 responses, would be associated with lower T_Reg_ percentages. Furthermore, we hypothesised that those with lower T_Reg_ percentages would have worse physical and mental health status and more depressive symptoms.

## Method

### Participants

This analysis was carried out on data collected from a subsample of the Heart Scan Study, an investigation of psychophysiological processes in cardiovascular disease (Hamer et al. 2010; Steptoe et al. 2011). Stress testing was carried out from 2006 to 2008. Blood samples were drawn from a subsample 3 years later as part of a study of cell stress proteins (Kaiser et al. 2014). All participants were members of the Whitehall II cohort, an ongoing study examining demographic, psychosocial and biological risk factors for coronary heart disease (Marmot et al. 1991). Participants were of white European origin and were aged 53–76 years. Inclusion criteria comprised no history or signs of CHD, no previous diagnosis or treatment for hypertension, diabetes, allergies or inflammatory diseases. Participants with a history of major depression or using antidepressant medication in the 12 months prior to testing were excluded. Out of those who underwent psychophysiological testing in the Heart Scan Study, 133 participants were selected for analysis of T_Reg_ percentages. Twelve of these individuals had missing data on the main factors included in these analyses (i.e. IL-6 and cortisol stress responses, questionnaire data), leaving a final sample of 121. All participants gave full informed consent to participate in the study, and ethical approval was obtained from the National Research Ethics Service.

### Psychophysiological testing at baseline

Psychophysiological testing was carried out in either the morning or the afternoon in a light and temperature-controlled laboratory and was based on a protocol previously described and used in this laboratory (Steptoe et al. 2002b). On arrival to the laboratory, participants were fitted with an intravenous line and allowed to rest for 30 min so that the participant could adapt after the insertion of the line. Following the rest period, a baseline blood (IL-6) and saliva (cortisol) sample was taken. Two behavioural tasks, designed to induce mental stress, were then administered in random order. The tasks were computerised versions of the Stroop task and mirror tracing. These tasks were selected because they have been shown to stimulate cardiac responses (Steptoe et al. 2002a) and individual responses to these tasks have been shown to predict increases in blood pressure and hypertension longitudinally (Brydon and Steptoe 2005) and subclinical atherosclerosis (Hamer et al. 2010). The tasks each lasted 5 min after which participants rested for 75 min. Both blood and saliva samples were taken immediately, 45 and 75 min after the stress protocol ended. A saliva sample was taken at 20 min post-protocol also.

### Biological stress measures

#### Plasma IL-6 analysis

Blood samples were collected in EDTA tubes and centrifuged immediately at 2500 rpm for 10 min at room temperature. Plasma was removed from the tubes and aliquoted into 0.5-mL portions and stored at − 80 °C until analysed. Plasma IL-6 was assayed using a Quantikine® high sensitivity two-site enzyme-linked immunosorbent assay (ELISA) from R&D Systems (Oxford, UK). The sensitivity of the assay ranged from 0.016 to 0.110 pg/mL and the intra-and inter-assay coefficient of variations (CV) were 7.3 and 7.7 %, respectively. IL-6 change scores were created by subtracting baseline IL-6 values from IL-6 at 75 min post-stress, in order to give a measure of the magnitude of the IL-6 stress response.

#### Salivary cortisol analysis

The saliva samples were collected using salivettes (Sarstedt, Leicester, UK). Levels of cortisol were determined using a time resolved immunoassay with fluorescence detection at the University of Dresden. Peak responses in cortisol tended to occur 20 min after the tasks; thus, a stress response change score was created by subtracting baseline cortisol values from those at 20 min after the tasks. Participants were then characterised as responders if a notable increase in cortisol was detected (≥1 nmol/L) as done before (Seldenrijk et al. 2012)

### Measurement of regulatory T cell percentage

#### PBMC and plasma isolation

Peripheral blood mononuclear cells (PBMCs) were isolated from 30 mL of heparinised venous blood by density centrifugation over Lymproprep (Axis Shield, Dundee, Scotland, UK) and washed two times with PBS (GE Healthcare, Pasching, Austria).

#### Regulatory and responder T cell percentage staining process

T_Reg_ (CD4 + CD25 + CD127^Low^) and responder T cell percentage (CD4 + CD25-CD127^High^) staining was performed on fresh whole blood. One hundred microlitres of whole blood from each participant was incubated with fluorescent anti-human CD4, CD25 and CD127 antibodies at room temperature (RT) for 20 min in the dark. After that, 1 mL of 1× lysing solution (BD, NJ, USA) was added and cell suspensions were incubated at room temperature for 15 min. The cell suspension was centrifuged at 300 *g* for 7 min. The supernatant was discarded, and the cell suspension was washed with 1× PBS + 0.1 % sodium azide before being fixed with 100 μL of 2 % paraformaldehyde (Alpha Aesar). The number of cells binding to each antibody was measured using flow cytometry with a FACSCalibur using appropriate settings. Twenty-thousand events in the lymphocyte gate were recorded for each sample and analysed using FlowJo software. Then, events from the lymphocyte gate were plotted in a CD4 dot plot. CD4+ cells were further subgated and plotted in CD25 versus CD127 dot plots. T_Reg_ percentage was defined as cells that express high levels of the CD25 molecule and low levels of CD127 while cell populations with high levels of the CD127 molecule and low levels of CD25 molecules were defined as responder T cells. Anti-CD25 antibodies (eBioscience) were purchased from Myltenyi Biotech (Gladbach, Germany), anti-CD127 FiTC from eBioscience, and anti-CD4 (eBioscience) was purchased from Becton Dickinson (New Jersey, USA).

### Measurement of psychological and physical well-being

We measured health status using the Short Form 36 Health Survey (SF-36) (Ware and Sherbourne 1992) at the follow-up visit 3 years after psychophysiological testing. The SF-36 is a 36-item measure of health-related quality of life that consists of eight subscales used to measure elements of physical and mental health. Physical and mental composite scores are computed from the eight subscales and range from 0 (worst possible health) to 100 (best possible health), with a normative value of 50. It has been used widely in older adults and is recommended for use where a detailed and broad-ranging health assessment is required (Haywood et al. 2005).

The Center for Epidemiologic Studies Depression (CES-D) Scale (Radloff 1977) was used to measure depressive symptomatology in the study sample. The CES-D Scale is a 20-item self-report measure composed of four distinct factors: depressive effect, somatic symptoms, interpersonal relations and positive affect. Respondents choose from four possible responses in a Likert format where ‘0’ is rarely or none of the time and ‘4’ is almost or all of the time (5–7 days). Scores range from 0 to 60 with higher scores reflecting greater levels of depressive symptoms.

### Other variables

A number of factors that are potentially relevant to associations between T_Reg_ percentage and other variables were measured as covariates. Sociodemographic characteristics included age, sex and socioeconomic status (indexed by employment grade). Body mass index (BMI) was calculated from height and weight recorded at the time of psychophysiological stress testing. Smoking status was also recorded at the time of psychophysiological stress testing. We included time of testing (morning/afternoon) as a covariate in analyses relating to IL-6 and cortisol stress responses. Time of testing was treated as a categorical variable as testing sessions only took place in the morning or the afternoon at a fixed time.

### Statistical analyses

All variables were normally distributed. Multiple linear regressions were used to examine the associations between acute biological stress responsivity, T_Reg_ percentage, and psychological and physical well-being. In regressions modelling associations between biological stress responsivity and T_Reg_ percentage measured 3 years later, T_Reg_ percentage was included as the dependent variable. In regressions modelling cross-sectional associations between T_Reg_ percentage and psychological and physical well-being, the well-being outcome measures were treated as dependent variables. Age, sex, employment grade, BMI and smoking status were included as covariates in all regressions. Time of testing (morning/afternoon) was included as an additional covariate in regressions that were modelling associations between cortisol and IL-6 responses to acute stress. The IL-6 stress response variable was modelled as a change score. Baseline IL-6 was included as a covariate in regressions examining the association between IL-6 stress responses and T_Reg_ percentage to assess the relative contribution of baseline and stress induced changes. We present results as both standardised and unstandardised regression coefficients. The significance level was set to *p* < 0.05 for all analyses, with precise *p* values reported for all test results. All analyses were conducted using SPSS version 20 (SPSS Inc., Chicago, IL, USA).

## Results

### Sample characteristics

The mean age of the current sample (*N* = 121) was 63.5 ± 5.7 years, and 30.6 % were male. Characteristics of the study population are provided in Table 1. All demographic measures, cortisol and IL-6 stress responses were recorded at baseline. T_Reg_ percentage, physical health status, mental health status and depressive symptoms were measured approximately 3 years later at a follow-up visit. There were no associations between T_Reg_ percentage and any of the demographic measures.

**Table 1:** Characteristics of the study population (*N* = 121)

Variable	Mean (SD) or %
Demographics	
Age	63.5 (5.7)
Gender (male)	30.6
Employment grade	
High	24.8
Medium	41.3
Low	33.9
BMI	
Normal	43.0
Overweight	43.8
Obese	13.2
Smoking	
Never smoked	70.2
Ex-smoker	26.4
Current smoker	3.3
T_reg_ cells and biological stress responsivity	
Regulatory T cells (%)	7.4 (2.1)
IL-6 at 75 min (pg/mL)	1.76 (1.11)
IL-6 baseline (pg/mL)	1.34 (0.82)
Cortisol responders, *n* (%)	66 (54.5 %)
Psychological and physical well-being	
Physical health status	79.0 (15.6)
Mental health status	77.4 (15.3)
Depressive symptoms	7.2 (6.8)

### Biological stress responses and regulatory T cells

There was a positive association between IL-6 levels at 75 min and T_Reg_ percentage 3 years later (B = 0.600, CI = 0.069–1.131, *p* = 0.027) after controlling for covariates including baseline IL-6. More pronounced IL-6 responses 75 min post-stress were related to higher T_Reg_ percentages. In contrast with the IL-6 results, cortisol responses to stress showed an inverse association with T_Reg_ percentages (B = −1.149, CI = −1.930–0.368, *p* = 0.004) after adjusting for covariates, in that those with blunted cortisol responses to acute stress were more likely to have a higher percentage of T_Reg_ cells. In order to confirm that the associations between biological stress responses and T_Regs_ were not the result of a staining artefact, we also measured associations between biological stress responses and responder T cells. In support of the associations reported above, we found significant associations between biological stress responses and responder T cell percentages in the opposite direction. IL-6 levels at 75 min post-stress were negatively associated with responder T cell percentage (B = −0.649, CI = −1.174–0.124, *p* = 0.016) after controlling for covariates including baseline IL-6 levels. This suggests that more pronounced IL-6 responses 75 min after stress were associated with lower percentages of responder T cells. In contrast, cortisol responses to stress were positively associated with percentages of responder T cells (B = 1.238, CI = 0.466–2.010, *p* = 0.002) after adjusting for covariates, suggesting that those with blunted cortisol responses had lower percentages of responder T cells.

### Regulatory T cells and psychological and physical well-being

The results displayed in Table 2 show the cross-sectional associations between T_Reg_ percentage and levels of physical and psychological well-being. T_Reg_ percentage was significantly associated with both worse physical (*t* = −2.047, *p* = 0.043) and mental (*t* = −2.709, *p* = 0.008) health status after controlling for covariates. Percentages of T_Reg_ cells were associated with scores on the CES-D (*t* = 3.131, *p* = 0.002) showing that higher proportions of T_Reg_ cells associated with higher depressive symptoms. We created a binary T_Reg_ variable using a median split and carried out age- and sex-adjusted ANCOVAs to assess the differences in the physical and psychological well-being measures. By way of illustration, the mean physical health status score adjusted for age and sex for people above the median in T_Reg_ percentage was 8.97 % lower than that for participants scoring below the median. There was a similar difference for mental health status scores (10.75 %), while depressive symptoms on the CESD were 63.02 % higher in the high than low T_Reg_ groups.

**Table 2:** Multiple regression analyses of regulatory T cell percentage on physical and psychological well-being

	Regulatory T cell percentage				
Dependent variable	B	SE	95 % CI	*β*	*p*
Physical health status (SF-36)	−1.402	0.685	−2.758–0.045	−0.190	**0.043**
Mental health status (SF-36)	−1.777	0.656	−3.076–0.477	−0.246	**0.008**
Depressive symptoms (CES-D)	0.907	0.290	0.333–1.480	0.284	**0.002**

Covariates: age, sex, BMI, smoking status, employment grade. Bold font indicates *p* < 0.05

We also examined associations between the percentage of responder T cells and the physical and psychological well-being variables. In support of the associations reported above, we found significant associations between percentages of responder T cells and physical and psychological well-being in the opposite directions. The percentage of responder T cells was significantly associated with both physical (B = 1.485, CI = 0.094–2.875, *p* = 0.037) and mental health status (B = 1.912, CI = 0.583–3.241, *p* = 0.005) after controlling for covariates, in that those with higher percentages of responder T cells had better physical and mental health. Percentages of responder T cells were negatively associated with scores on the CES-D (B = −0.965, CI = −1.552–0.378, *p* = 0.001) showing that lower proportions of responder T cells were associated with more depressive symptoms.

## Discussion

We hypothesised that dysregulated biological responses to acute psychosocial stress, i.e. a blunted cortisol response and an exaggerated IL-6 response, would be associated with lower T_Reg_ percentages. We also hypothesised that individuals with a lower percentage of T_Regs_ would have worse physical and mental health status and more depressive symptoms. Contrary to prediction, our results were the opposite of our hypotheses. Rather than a lower percentage of T_Regs_, we found that both blunted cortisol responses and exaggerated IL-6 responses to acute stress in normal healthy volunteers were associated with a higher percentage of circulating T_Regs_, after controlling for covariates. We also showed that elevated T_Reg_ percentage was cross-sectionally associated with worse physical and mental health status, and higher levels of depressive symptomatology. Taken together, these results suggest that dysfunctional biological responses to acute psychosocial stress are associated with a subsequent higher T_Reg_ percentage, which in turn is also associated with poorer health and well-being.

To our knowledge, this is the first study to show an association between biological stress responsivity and T_Reg_ disturbance. Although these measures were taken 3 years apart, we did not measure T_Reg_ percentage at the time of stress testing, so we do not know whether the same pattern would have been present at that time. However, the T_Reg_ cell lineage has been shown to be largely stable over time both in vitro and in vivo (Rubtsov et al. 2010; Hori 2011). This stability is a prerequisite which allows T_Regs_ to limit transient tissue damage in the face of inflammation (Sakaguchi et al. 2013).

Our results are in line with studies that have shown that prolonged exposure to stress in healthy people is associated with increases in T_Reg_ percentages. For example, T_Reg_ percentages increase in students undergoing a period of examination stress (Höglund et al. 2006). Similarly, an increase in T_Reg_ numbers has also been seen in murine models of chronic stress (Saul et al. 2005; Kim et al. 2012). Blunted cortisol responses and exaggerated IL-6 responses to acute stress are associated with chronic stress (Hamer et al. 2006; Elzinga et al. 2008; Carpenter et al. 2010; Goldman-Mellor et al. 2012). This may be why we see increased T_Reg_ percentages in members of our sample with dysregulated responses to acute stress, as these responses may be serving as a proxy for chronic stress. In order to lend support to our findings, we also examined associations between biological stress responses and percentages of CD4+ responder T cells. T_Regs_ induce apoptosis in, and suppress proliferation of CD4+ responder T cells (Sakaguchi et al. 2009). We found associations between percentages of responder T cells and biological stress responses, as well as physical and psychological well-being, but in the opposite directions to those found with T_Regs_. Therefore, these results with responder T cells strengthen the associations reported between biological stress responses and T_Regs_ and confirm that results are not due to an artefact of the T_Reg_ staining process.

A study by Freier et al. (2010) indicated that, cross-sectionally, acute stress induced a transient decrease in T_Reg_ cells, which may serve to facilitate the mounting of an inflammatory response. However, chronic situations of psychological stress in healthy individuals might lead to increases in T_Reg_ percentages, having the effect of counteracting the heightened levels of cytotoxic T cells in the periphery and the increased levels of inflammation characteristic of chronic stress (Dhabhar 2009).

Cross-sectionally, we found that T_Reg_ percentages were associated with poorer self-reported health status in healthy individuals. This result supports the idea that increased T_Reg_ numbers may be indicative of greater risk of future disease development. As noted in the “Introduction”, a number of stress-related conditions are associated with a decreased T_Reg_ percentage. It is thus possible that our results indicate that people who have dysregulated biological responses to acute stress, but are still healthy, may be in a state of threatened homeostasis as shown by increased percentages of T_Regs_.

Healthy individuals have a range of mechanisms to maintain immune homeostasis of which T_Regs_ are included. T_Reg_ suppression is mediated by both cell contact and a number of inflammatory cytokines (Beissert et al. 2006). Pukhalsky et al. (2008) suggest that as inflammation increases after prolonged stress, T_Regs_ could accumulate over time in order to counter this exaggerated inflammatory stress response. Research has shown that a more prolific history of infection and illness over the lifespan is associated with higher percentages of T_Regs_ in those aged 65 and above (Trzonkowski et al. 2006). This may be why we have seen increased T_Regs_ in those with dysfunctional biological responses to acute stress and in those with poor health status in our sample. It is clear, however, that increased numbers of T_Regs_ cannot be sustained for long as many stress-related disorders are characterised by a decrease in T_Reg_ levels. The switch from chronic stress to a stress-related disease may be mediated in part by this change in T_Reg_ levels. It has been demonstrated in vitro that T_Regs_ lose function when incubated with IL-6 (Pasare and Medzhitov 2003). Later murine work then demonstrated that this decrease in function was probably due to loss of FoxP3—the transcription factor necessary for T_Reg_ expression—in the face of inflammation (Zhou et al 2009). When T_Regs_ lose this transcription factor they become ‘exT_Regs_’ which are effector-like T cells that no longer serve to regulate immune function (Zhou et al. 2009). This may be one mechanism that mediates this shift in T_Reg_ levels from chronic stress to stress-related disease.

The strength of the current study is that we chose to assess the CD4 + CD25 + CD127^Low^FoxP3+ natural T_Reg_ subtype. FoxP3 is the transcription factor that guides naive CD4 cells towards the T_Reg_ variety (Naugler and Karin 2008). CD127 is the receptor for IL-7 and is found to be inversely correlated with FoxP3 (Liu et al. 2006). A combination of CD4, CD25, CD127 and FoxP3 allows for a highly purified measure of T_Reg_ cell percentage (Liu et al. 2006). Many studies examining the role of T_Regs_ in stress-related diseases report CD4 + CD25+ T_Reg_ levels only. CD25 is the IL-2 receptor which is known to be expressed across a continuum of cells (Brusko et al. 2005) meaning that measuring CD4 + CD25+ cells may not provide a precise assessment of T_Reg_ numbers. A further strength of this study was the use of well-validated measures of physical and mental well-being and depressive symptomatology.

As well as strengths, the study has a number of limitations. T_Reg_ percentage was measured 3 years after biological stress responses were assessed. Although the reported associations were temporal in nature, we cannot infer causality as it was beyond the scope of the study to control for baseline T_Reg_ percentage. However, the T_Reg_ cell lineage has been shown to be largely stable over time both in vitro and in vivo (Rubtsov et al. 2010; Hori 2011). Secondly, the study involved healthy older men and women of white European origin. Thus, the pattern of results may not be generalised to other groups.

T_Regs_ play an important role in the stress-disease process (Miller 2010). This study has demonstrated that dysfunctional neuroendocrine and inflammatory responses to acute psychosocial stress are associated with an elevated T_Reg_ profile, and that this profile is associated with poorer health. We posit that T_Regs_ act as one of the defences against elevated inflammation and therefore may serve as a harbinger of future stress-related illness. It is possible that in the future, an increased T_Reg_ profile could be used in clinical settings to identify those at greatest risk of disease.
